# High‐Intensity Interval Training and Moderate‐Intensity Continuous Training on Ventricular‐Arterial Coupling (VAC) in Young Women With Obesity

**DOI:** 10.1111/echo.70355

**Published:** 2025-11-19

**Authors:** Caroline Ferraz Simões, Rogério Toshiro Passos Okawa, João Carlos Locatelli, Gustavo Henrique de Oliveira, Higor Barbosa Reck, Lucimere Bohn, Jorge Mota, Wendell Arthur Lopes

**Affiliations:** ^1^ Research Group on Systemic Arterial Hypertension Arterial Stiffness and Vascular Aging Maringa Brazil; ^2^ Cesumar University Maringa Brazil; ^3^ Department of Medicine State University of Maringa Maringa Brazil; ^4^ School of Human Sciences (Sport Science, Exercise and Health) The University of Western Australia Perth Western Australia Australia; ^5^ Faculty of Sport University of Porto Porto Portugal; ^6^ Research Centre for Physical Activity Health and Leisure Porto Portugal; ^7^ Department of Physical Education State University of Maringa Maringa Brazil

**Keywords:** aerobic training, arterial elastance, global longitudinal strain, overweight, pulse wave velocity, ventricular elastance

## Abstract

**Aim:**

To investigate the effects of an 8‐weeks of high‐intensity interval training (HIIT) or moderate‐intensity continuous training (MICT) program on ventricular‐arterial coupling (VAC) in young women with obesity.

**Methods:**

Twenty‐four obese women completed an 8‐week supervised aerobic training program (3 sessions/week) assigned to either HIIT (*n* = 11) or MICT (*n* = 13). The HIIT protocol involved four 4‐min bouts at 85%–95% of the maximum heart rate (HR_max_), while the MICT consisted of continuous walking/running for 41 min at 65%–75% of HR_max_. VAC was assessed using two methods: (1) the ratio of pulse wave velocity to global longitudinal strain (PWV/GLS), and (2) the ratio of arterial to ventricular elastance (E_a_/E_es_).

**Results:**

Both HIIT (−0.35 ± 0.01 to −0.31 ± 0.05 m/s%; *p* = 0.005) and MICT (−0.35 ± 0.01 to −0.30 ± 0.05 m/s%; *p* = 0.003) significantly improved the PWV/GLS ratio. However, only HIIT led to a significant reduction in the E_a_/E_es_ ratio (0.88 ± 0.07 to 0.80 ± 0.09 mmHg/mL; *p* = 0.024), with a significant correlation between relative changes in PWV/GLS and E_a_/E_es_ ratio (*r* = 0.749; *p* = 0.008).

**Conclusions:**

Both HIIT and MICT improved VAC as assessed by PWV/GLS ratio in young obese women. In contrast, elastance‐derived improvements (E_a_/E_es_) were observed only following HIIT. These findings suggest that PWV/GLS may serve as a more sensitive and integrative marker for detecting exercise‐induced improvements in VAC.

## Graphical abstract

Aerobic training, HIIT and MICT, proved effective in improving VAC in young women with obesity as measured by the PWV/GLS ratio.

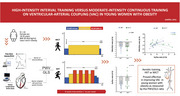

## 1 Introduction

Over the last decades, obesity has reached pandemic levels, being considered one of the main public health problems nowadays and an important predictor for cardiovascular diseases [1]. Substantial evidence indicates that excessive adipose tissue adversely affects the cardiovascular system through both direct and indirect mechanisms [2]. Moreover, obesity has been shown to impair the interaction between cardiac and vascular function, known as ventricular‐arterial coupling (VAC) [3]. Previous studies have identified body mass index (BMI) as a significant predictor of increased arterial elastance (E_a_) and/or ventricular elastance (E_es_), which leads to an elevated E_a_/E_es_ ratio, an established but indirect method for assessing VAC [3].

Given the methodological limitations and complexity associated with elastance‐based assessments, alternative approaches have been proposed that incorporate more sensitive markers of vascular and myocardial function [4]. Ikonomidis et al. [5] introduced a novel VAC assessment method using the ratio of pulse wave velocity (PWV) to global longitudinal strain (GLS). This PWV/GLS ratio has shown significant association with subclinical cardiovascular impairment, such as increased carotid intima‐media thickness, reduced coronary flow reserve, and diastolic dysfunction in hypertensive patients, associations not observed with the traditional elastance method [5]. Furthermore, this ratio has been sensitive to therapeutic interventions in populations with type 2 diabetes [6] and rheumatoid arthritis [7].

Despite its potential, the effects of different aerobic training modalities on VAC assessed via PWV/GLS ratio remain largely unexplored. Prior studies using the elastance method have demonstrated that the aerobic exercise improves VAC [8], and other research has shown that such training can reduce PWV [9], and enhance GLS [10] across various populations. This supports the hypothesis that aerobic training may also enhance VAC when measured using the PWV/GLS ratio.

High‐intensity interval training (HIIT) has gained popularity due to its time‐efficiency nature and has shown comparable or superior adherence and enjoyment rates among individuals with overweight or obesity when compared to the moderate‐intensity continuous training (MICT) [11]. Additionally, HIIT has been found to produce greater improvements in vascular function relative to MICT [12, 13, 14]. However, whether HIIT is superior in enhancing the PWV/GLS ratio, and thus VAC, has not yet been fully elucidated.

Therefore, the aim of this study was to investigate the effects of 8 weeks of HIIT and MICT on VAC, assessed by two different methods in young women with obesity. A secondary objective was to examine whether changes in this novel index correspond with those derived from the traditional elastance‐based method.

## 2 Materials and Methods

### 2.1 Study Population

A total of 44 women were enrolled in this randomized controlled study and randomly assigned to perform either HIIT (*n *= 22) or MICT (*n *= 22). Both exercise interventions were conducted three times per week over 8 weeks, totaling 24 sessions.

The inclusion criteria consisted of: (1) female sex; (2) age between 18 and 35 years; and (3) BMI between 30 and 39.9 kg/m^2^ (Class I and II obesity). The exclusion criteria were the following: (1) previous weight‐loss surgeries or participation in exercise or dietary interventions within the past 6 months; (2) classification as physically active according to the International Physical Activity Questionnaire [15]; (3) physical limitations that would prevent participation in the exercise protocols; (4) presence of endocrine, vascular, cardiac diseases or other cardiovascular risk factors aside from obesity; and (5) use of any medications.

All participants underwent a medical evaluation, including clinical history, physical examination, and cardiovascular screening tests (resting ECG, exercise treadmill test, transthoracic echocardiography, and carotid Doppler ultrasonography) to confirm eligibility and ensure cardiovascular safety.

Participants who met the inclusion criteria and provided written informed consent were instructed to maintain their usual dietary and lifestyle habits and to refrain from engaging in any additional physical activity during the study period.

This study was approved by the local ethics committee (protocol number: 08935419.5.0000.0104) and registered on the Brazilian Clinical Trials Registry (RBR‐3v3dqf). All procedures complied with the Declaration of Helsinki and Resolution 466/2012 of the Brazilian National Health Council.

### 2.2 Exercise Protocols

Both exercise protocols consisted of walking/running, according to the individual maximum heart rate (HR_max_). The HIIT protocol applied starts with a 10‐min warm‐up, followed by four bouts of 4 min each at 85%–95% of the HR_max_, interspersed by 3‐min active recovery periods. The protocol ends with a 5‐min cool down, lasting 40 min. The MICT protocol starts with a 5‐min warm‐up, followed by 41 min at 65%–75% of HR_max_, ending with a 2‐min cool‐down, with a total of 48 min. Both protocols were standardized to be isocaloric [13]. HR was continuously monitored with a Polar H10 sensor. HR data were recorded via Polar Team software on an iPad (Apple, CA, USA). Verbal encouragement was provided when necessary to maintain the target intensity.

### 2.3 Echocardiographic and Pulse Wave Velocity Assessments

Left ventricular function was assessed via a transthoracic echocardiogram using a commercially available ultrasound system (GE Healthcare, Norway) using a 1.4–4.6 MHz XD transducer, performed by an experienced and accredited sonographer, who was blinded to group allocation. Participants were positioned on an echocardiographic examination bed in the semi‐recumbent left lateral position, and echocardiographic images were obtained at end expiration. Left ventricular end‐diastolic volume (EDV) and end‐systolic volume (ESV) were assessed using the Simpson's biplane method, using mean values from the four‐ and two‐chamber apical views. Stroke volume (SV) was calculated as the difference between EDV and ESV. GLS was assessed by 2D speckle tracking echocardiography. Apical four, three, and two‐chamber views were obtained, and the mean values from the 18 segments derived from these views were used to calculate GLS. When it was not possible to obtain apical two and/or three chamber views, a mean value from the six segments obtained from the apical four‐chamber view was used to calculate GLS. The echocardiographic assessments were in accordance with comprehensive guidelines [16]. Noteworthy, regarding GLS, more negative values represent greater deformation of the longitudinal myocardial fibers and can be interpreted as enhanced systolic function [16]. Intraobserver variability was performed on 10 individuals, randomly selected from the study sample. GLS (3.0%), EDV (6.4%), and ESV (7.9%) measurements showed a good coefficient of variation.

PWV was assessed using the SphygmoCor XCEL system (AtCor Medical, Sydney, Australia), which measures carotid‐femoral PWV through applanation tonometry and volumetric cuff‐based pulse detection. PWV was calculated as the distance between carotid and femoral sites divided by pulse transit time (expressed in m/s) [17]. Hemodynamic measurements were taken twice, wherein a third measurement was performed when the difference between the first two measurements was superior to 5 mmHg, or when the measurements failed the automated software‐integrated quality check.

### 2.4 Ventricular‐Arterial Coupling Assessment

In this study, VAC was estimated non‐invasively using two methods: (1) as the ratio between carotid‐femoral PWV and GLS (PWV/GLS ratio), (2) as the ratio between E_a_ and E_es_ (E_a_/E_es_ ratio). E_a_ was calculated as the ratio between end‐systolic blood pressure (ESP) and SV (E_a _= ESP/SV), with ESP estimated by multiplying systolic blood pressure (SBP) by 0.9 (ESP = SBP × 0.9). E_es_ was calculated by the ratio between ESP and end systolic volume (ESV) subtracting V0, assuming that volume intercept is negligible compared with ESV (E_es _= ESP/ESV‐V0) [4].

### 2.5 Complementary Assessments

All echocardiographic measurements followed the American Society of Echocardiography guidelines [16]. Body composition was assessed using a bioelectrical impedance (Maltron BF‐906, Rayleigh, UK). Body mass and height were measured using a calibrated mechanical scale with a stadiometer (Filizola, Sao Paulo, Brazil). Blood pressure was measured using a validated automatic device (Omron HEM 7320, São Paulo, Brasil). Cardiorespiratory fitness was assessed via a graded ramp treadmill test (Micromed, Centurion 300, Brasília, Brazil) with peak oxygen consumption (VO_2peak_) measured by open‐circuit spirometry using a gas analyzer (Metalyzer, Cortex, USA).

### 2.6 Data Analysis

All data were expressed as mean ±and standard deviation. Two‐way repeated‐measures ANOVA was used to assess group (HIIT and MICT) × time (pre vs. post) interactions. Bonferroni corrections were applied for multiple comparisons. Effect sizes were calculated using Cohen's d. Post hoc power analysis (GPower 3.1.9.7) indicated a statistical power >80% for the observed sample size. Pearson's correlation coefficient was used to assess associations between changes in PWV/GLS and E_a_/E_es_ ratios. A significance level of *p* ≤ 0.05 was adopted. All analyses were performed using SPSS version 23 (IBM, NY, USA).

## 3 Results

**FIGURE 1:**
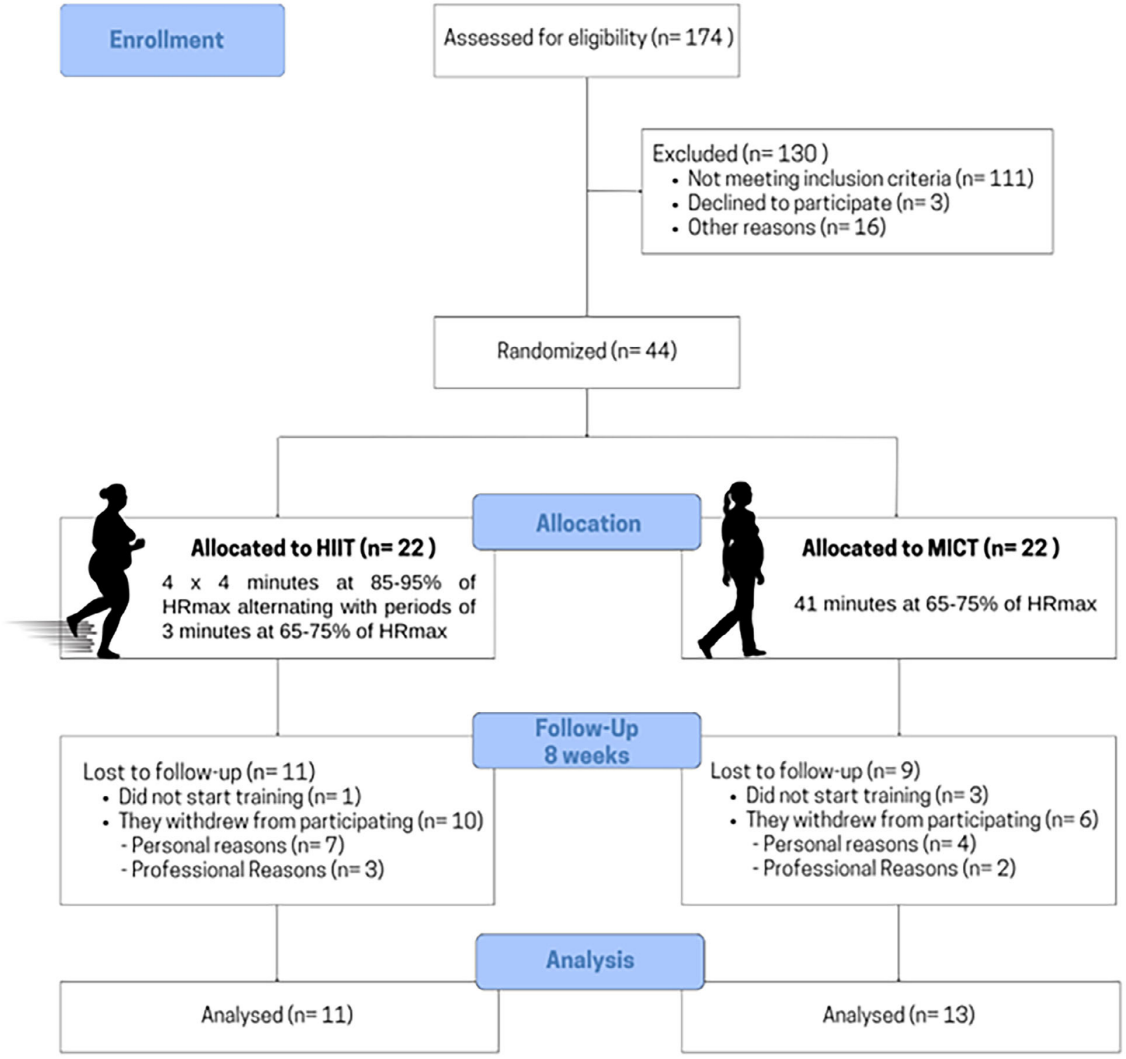
Flowchart of study participants.

A total of 24 women completed all assessments and the 8‐week intervention (HIIT: *n* = 11; MICT: *n* = 13) (Figure 1). Baseline characteristics of both groups are presented in Table 1, with no significant differences between groups for any variable. Target HR was consistently achieved during training sessions in both HIIT (92 ± 2% HR_max_) and MICT (73 ± 2% HR_max_) groups.

**TABLE 1:** Baseline sample characteristics.

Characteristic	HIIT (*n* = 11)	MICT (*n* = 13)	*p*
**Age and anthropometrics**			
Age [years]	29.5 ± 4.9	27.1 ± 5.1	0.248
Body weight [kg]	90.8 ± 14.1	95.4 ± 6.2	0.276
BMI [kg/m^2^]	34.1 ± 3.9	35.7 ± 2.4	0.221
Body fat [%]	44.1 ± 3.6	44.8 ± 2.2	0.510
**Hemodynamics and cardiorespiratory fitness**			
SBP [mmHg]	128.3 ± 12.8	122.6 ± 10.3	0.229
DBP [mmHg]	76.8 ± 11.1	74.8 ± 5.8	0.573
Resting HR [beats/min]	78.0 ± 9.8	79.4 ± 10.2	0.727
VO_2peak_ [mL/min/kg]	27.5 ± 3.7	26.9 ± 3.6	0.308
**Echocardiography**			
LV mass index [g/m^2^]	120.0 ± 19.9	126.6 ± 5.8	0.355
SV [mL]	41.8 ± 5.7	42.5 ± 8.5	0.801
LVEDV [mL]	78.6 ± 10.1	80.1 ± 13.2	0.758
LVESV [mL]	36.9 ± 4.9	37.6 ± 5.6	0.730

Abbreviations: BMI, body mass index; DBP, Diastolic blood pressure; HIIT, high‐intensity interval training; HR, heart rate; LVEDV, left ventricle end‐diastolic volume; LVESV, left ventricle end‐systolic volume; MICT, moderate‐intensity continuous training; SBP, systolic blood pressure; SV, left ventricle stroke volume; VO_2peak_, peak oxygen consumption.

Regarding anthropometric, body composition and cardiorespiratory fitness, only the HIIT group showed significant improvements after 8 weeks in body weight (90.7 ± 14.07 to 88.8 ± 13.05 kg, *p* = 0.002), BMI (34.1 ± 3.9 to 33.4 ± 3.5 kg/m^2^, *p* = 0.002), and body fat percentage (44.1 ± 3.6 to 41.2 ± 3.7%, *p* < 0.001), as well as an increase in VO_2peak_ (27.5 ± 3.7 to 29.4 ± 4.9 mL/kg/min, *p* = 0.024).

Table 2 displays the mean ± standard deviations and absolute changes for PWV/GLS and E_a_/E_es_ ratios, along with their respective components, before and after the interventions. Both HIIT (−0.35 ± 0.07 to −0.31 ± 0.05 m/s%, *p* = 0.005) and MICT (−0.35 ± 0.07 to −0.30 ± 0.04 m/s%, *p* = 0.003) significantly improved the PWV/GLS ratio. However, E_a_/E_es_ ratio showed a significant reduction only following HIIT (−0.88 ± 0.07 to 0.80 ± 0.09 mmHg/mL, *p* = 0.024), with no meaningful change observed in the MICT group. SV increased significantly in both groups (HIIT: Δ = 5.59 ± 3.1, *p* < 0.001 and MICT: Δ = 5.39 ± 4.2, *p* < 0.001), whereas ESP decreased only in HIIT (Δ = 5.65 ± 3.9, *p* = 0.004).

**TABLE 2:** Mean values, standard deviations, and absolute changes of PWV/GLS and E_a_/E_es_ ratios and their components before and after HIIT and MICT interventions.

	HIIT					MICT					
	Pre	Post	Δ	ES	*p*	Pre	Post	Δ	ES	*p*	Time × Group
**Variables related to E_a_/E_es_ ratio**											
ESP [mmHg]	115.4 ± 9.1	109.8 ± 9.2	−5.65 ± 3.9	0.65	0.004	110.3 ± 6.9	107.7 ± 5.6	−2.64 ± 7.1	0.19	0.343	0.376
SV [mL]	41.8 ± 5.7	47.4 ± 3.9	5.59 ± 3.1	1.19	<0.001	42.5 ± 8.5	47.9 ± 8.1	5.39 ± 4.2	0.68	<0.001	0.912
E_a_ [mmHg∕mL]	2.8 ± 0.3	2.3 ± 0.3	−0.46 ± 0.3	1.55	0.001	2.7 ± 0.5	2.4 ± 0.6	−0.31 ± 0.3	0.59	0.001	0.310
E_es_ [mmHg∕mL]	3.2 ± 0.3	2.9 ± 0.2	−0.23 ± 0.4	0.90	0.037	3.0 ± 0.5	2.7 ± 0.6	−0.30 ± 0.3	0.53	0.012	0.458
E_a_/E_es_ ratio [mmHg∕mL]	0.88 ± 0.07	0.80 ± 0.09	−0.09 ± 0.10	1.04	0.024	0.90 ± 0.12	0.89 ± 0.18	−0.01 ± 0.11	0.14	0.435	0.239
**Variables related to PWV/GLS ratio**											
PWV [m/s)	6.6 ± 0.8	6.2 ± 0.8	−0.37 ± 0.2	0.48	0.016	6.5 ± 0.8	6.2 ± 0.8	−0.35 ± 0.3	0.47	0.023	0.950
GLS [%]	−18.9 ± 2.4	−20.2 ± 2.0	1.37 ± 1.7	0.63	<0.001	−19.3 ± 2.8	−20.6 ± 1.9	1.26 ± 1.8	0.52	0.001	0.866
PWV/GLS ratio [m/s%]	−0.35 ± 0.07	−0.31 ± 0.05	0.04 ± 0.04	1.16	0.005	−0.35 ± 0.07	−0.30 ± 0.04	0.04 ± 0.05	1.44	0.003	0.871

Abbreviations: E_a_, arterial elastance; E_es_, ventricular elastance; ESP, end‐systolic blood pressure; GLS, global longitudinal strain; HIIT, high‐intensity interval training; MICT, moderate‐intensity continuous training; PWV, carotid‐femoral pulse wave velocity; SV, stroke volume.

**FIGURE 2:**
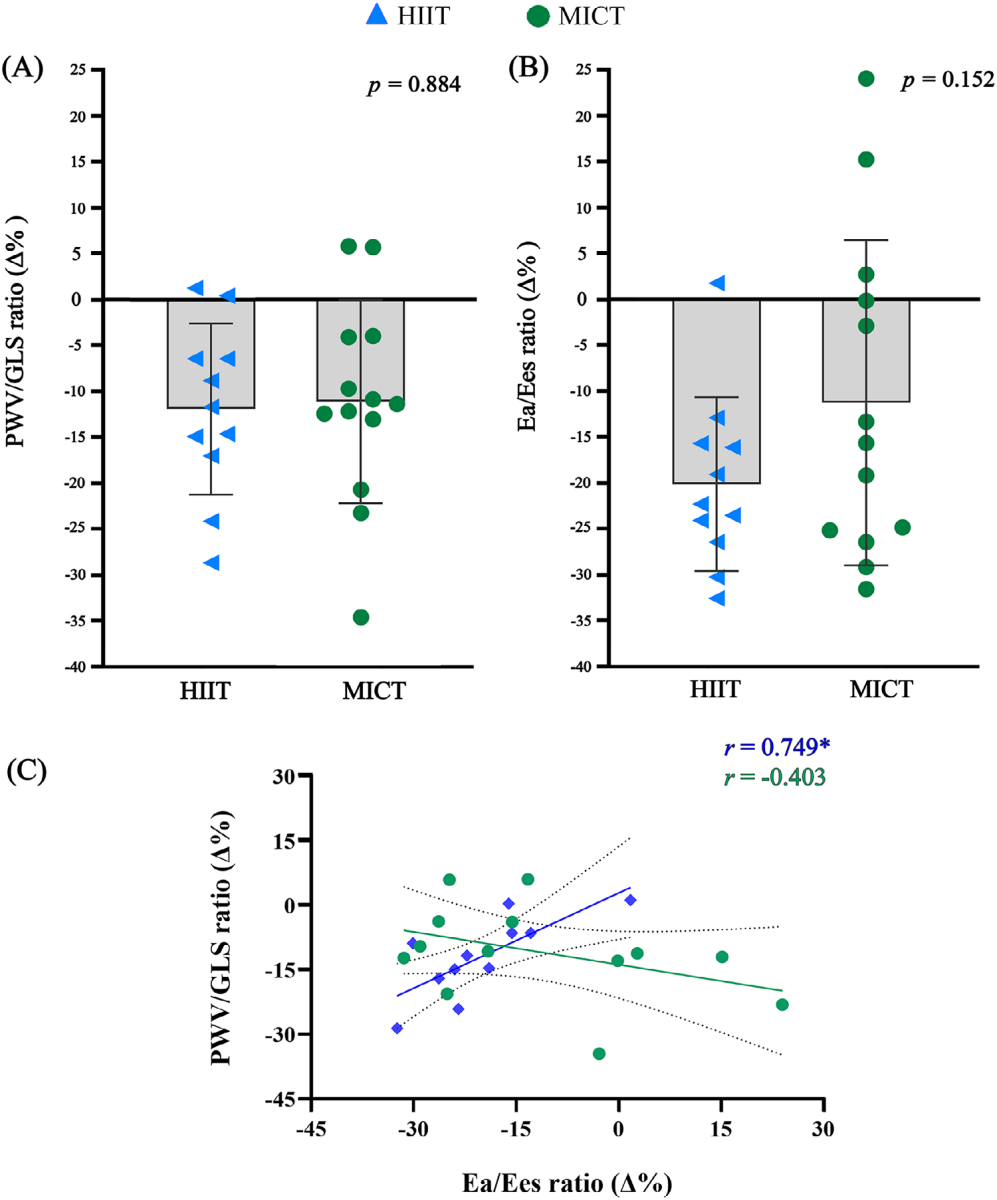
Relative changes after 8‐weeks of high‐intensity interval training (HIIT) and moderate‐intensity continuous training (MICT) in (A) PWV/GLS ratio, (B) E_a_/E_es_ ratio and (C) Correlation between PWV/GLS and E_a_/E_es_ ratio. *Statistical significance (*p* < 0.05).

Figure 2A,B illustrates the relative changes in PWV/GLS ratio (HIIT: %Δ = −11.9 ± 9.3 and MICT: %Δ = −11.1 ± 11.0; *p* = 0.884) and E_a_/E_es_ ratios (HIIT: %Δ = −20.0 ± 9.4 and MICT: %Δ = −11.2 ± 17.6; *p* = 0.152). Figure 2C shows the correlation between relative changes in PWV/GLS and E_a_/E_es_ ratio, which were statistically significant only for HIIT (*r* = −0.749, *p* = 0.008).

## 4 Discussion

Our findings indicate that both HIIT and MICT significantly improved the PWV/GLS ratio, suggesting enhanced VAC following aerobic training in young women with obesity. However, only the HIIT group exhibited a significant reduction in the E_a_/E_es_ ratio, accompanied by a significant correlation between relative changes in PWV/GLS and E_a_/E_es_ ratios. These results suggest that the PWV/GLS ratio may serve as a more sensitive and integrative marker for detecting exercise‐induced changes in VAC, even when traditional elastance‐based indices remain unchanged.

The PWV/GLS ratio has recently emerged as a promising, non‐invasive index for evaluating VAC, integrating vascular stiffness and myocardial deformation [4]. Previous studies have demonstrated that this ratio is independently associated with adverse cardiovascular outcomes in patients with heart failure with preserved ejection fraction (HFpEF) [18], and correlates more strongly with subclinical target organ damage than the E_a_/E_es_ ratio [19]. A higher PWV/GLS ratio reflects a mismatch between arterial load and myocardial contractility [20].

In our study, both aerobic training modalities led to a significant reduction in PWV/GLS ratio (Δ = −0.04 m/s.%), corresponding to an approximate 11% improvement in VAC, which reflects enhanced arterial compliance and myocardial efficiency [21]. Aerobic exercise enhances vascular compliance via multiple mechanisms, including increased nitric oxide bioavailability, reduced oxidative stress and inflammation, and downregulation of the renin‐angiotensin‐aldosterone system [9, 13, 22, 23, 24]. Concurrently, it improves left ventricular mechanics by reducing afterload and enhancing myocardial strain [10, 14], thereby optimizing ventricular‐arterial interaction [8].

In contrast, VAC improvements as assessed by the E_a_/E_es_ ratio were observed only in the HIIT group, mainly driven by a greater reduction in E_a_. HIIT induces stronger hemodynamic and shear stress compared to MICT, leading to enhanced endothelial function and reduced systemic vascular resistance [13, 14, 24]. These vascular adaptations lower E_a_ and improve VAC, consistent with previous findings showing superior vascular effects of HIIT [12]. Additionally, improvements in body composition and cardiorespiratory fitness after HIIT observed in this study may have contributed to reduced E_a_. Decreases in body weight and fat mass attenuate sympathetic activity and systemic vascular resistance, while increased VO_2peak_ enhances endothelial function and vasodilatory capacity [25]. Together, these changes reduce mean BP and afterload, promoting lower E_a_ and a more favorable VAC profile.

Despite its physiological significance, the elastance‐based method presents several limitations. E_a_ is strongly influenced by systemic vascular resistance and heart rate, which can introduce considerable variability [26]. Additionally, E_a_ does not distinguish between the resistive and pulsatile components of arterial load [26, 27], potentially limiting its sensitivity to subtle training‐induced vascular adaptations. Furthermore, both E_a_ and E_es_ are typically estimated non‐invasively using surrogate measures such as ESP and SV, which may reduce accuracy, particularly in individuals with altered hemodynamics [21, 27]. These limitations highlight the need for complementary or alternative indices such as the PWV/GLS ratio.

Interestingly, although baseline PWV/GLS ratio values in our cohort were below the proposed cut‐off for subclinical dysfunction in young women (–0.41 m/s%) [28], significant improvements were still observed after aerobic training protocols. This suggests the PWV/GLS ratio is sensitive to early cardiovascular adaptations even in the absence of overt disease, supporting its utility as a dynamic marker of functional improvement. Previous studies have also shown that this ratio correlates more strongly with subclinical target organ damage than the E_a_/E_es_ ratio [18, 19, 27, 29], further supporting its clinical relevance.

Moreover, the distinct patterns of correlation observed between relative changes in PWV/GLS and E_a_/E_es_ ratios imply that these indices may capture different physiological domains of VAC. While PWV reflects large artery stiffness and GLS assesses myocardial deformation, the E_a_/E_es_ ratio is a more mechanistic but less practical tool in clinical settings. In fact, a recent study reported a weak correlation between the two methods in healthy adults, likely due to E_a_ dependency on systemic vascular resistance and heart rate, which may account for up to 98% of its variability [27].

Despite its limitations, the elastance‐based method remains a valuable tool for understanding cardiovascular performance and energetic efficiency [8]. Nonetheless, our results support the use of PWV/GLS ratio as a more practical, reproducible, and clinically meaningful alternative for assessing exercise‐induced improvements in VAC.

### 4.1 Strengths and Limitations

To our knowledge, this is the first study to directly compare the impact of HIIT and MICT on VAC using the PWV/GLS ratio. Given that both exercise modalities are recommended by major international health organizations [30, 31], our findings support their efficacy in improving VAC in young women with obesity. Furthermore, the use of the PWV/GLS ratio provides a novel, non‐invasive, and sensitive tool for assessing integrated vascular and myocardial responses to aerobic training.

However, several limitations must be acknowledged. First, the sample consisted exclusively of young women without obesity‐related comorbidities, limiting generalizability to other populations such as obese men or individuals with cardiometabolic disease. In addition, the findings from the present study need to be interpreted with caution due to the small sample size. Second, due to the lack of prior studies investigating exercise‐induced changes in PWV/GLS ratio, our findings remain exploratory and lack external comparators. Finally, although we found a correlation between PWV/GLS and elastance‐derived assessments following HIIT, the elastance method itself is not a true gold standard. Future studies using validated invasive or imaging‐based reference techniques are needed to confirm the clinical relevance of PWV/GLS.

## 5 Conclusion

In conclusion, both HIIT and MICT effectively improved VAC in young women with obesity, as assessed by the PWV/GLS ratio. Moreover, the differing response patterns and correlations between PWV/GLS and elastance‐based measures suggest they may capture complementary but distinct physiological aspects of VAC adaptation to aerobic exercise. Taken together, our findings support the use PWV/GLS as a sensitive, reproducible, and clinically relevant tool for non‐invasive monitoring of cardiovascular adaptations induced by exercise training.

## Funding

This study was supported by a grant from the Fundação Araucária (CP 20/2018 PPP).

## Ethics Statement

This study was previously approved by the local ethics committee of the State University of Maringa (protocol number: 08935419.5.0000.0104).

## Conflicts of Interest

The authors declare no conflicts of interest.

## Data Availability

The data that support the findings of this study are available on request from the corresponding author. The data are not publicly available due to privacy or ethical restrictions.
